# Limited contribution of non-intensive chicken farming to ESBL-producing *Escherichia coli* colonization in humans in Vietnam: an epidemiological and genomic analysis

**DOI:** 10.1093/jac/dky506

**Published:** 2019-01-09

**Authors:** Vinh Trung Nguyen, Dorota Jamrozy, Sébastien Matamoros, Juan J Carrique-Mas, Huynh Mai Ho, Quoc Hieu Thai, Thi Nhu Mai Nguyen, Jaap A Wagenaar, Guy Thwaites, Julian Parkhill, Constance Schultsz, Thi Hoa Ngo

**Affiliations:** 1 Oxford University Clinical Research Unit, Centre for Tropical Medicine, Ho Chi Minh City, Vietnam; 2 Wellcome Sanger Institute, Cambridge, UK; 3 Department of Medical Microbiology, Amsterdam UMC, University of Amsterdam, Amsterdam, The Netherlands; 4 Department of Global Health-Amsterdam Institute for Global Health and Development, Amsterdam, The Netherlands; 5 Centre for Tropical Medicine, Nuffield Department of Medicine, University of Oxford, Oxford, UK; 6 Sub-Department of Animal Health, My Tho, Tien Giang, Vietnam; 7 Preventive Medicine Center, My Tho, Tien Giang, Vietnam; 8 Department of Infectious Diseases and Immunology, Faculty of Veterinary Medicine, Utrecht University, Utrecht, The Netherlands; 9 Central Veterinary Institute of Wageningen UR, Lelystad, The Netherlands

## Abstract

**Objectives:**

To investigate the risk of colonization with ESBL-producing *Escherichia coli* (ESBL-Ec) in humans in Vietnam associated with non-intensive chicken farming.

**Methods:**

Faecal samples from 204 randomly selected farmers and their chickens, and from 306 age- and sex-matched community-based individuals who did not raise poultry were collected. Antimicrobial usage in chickens and humans was assessed by medicine cabinet surveys. WGS was employed to obtain a high-resolution genomic comparison between ESBL-Ec isolated from humans and chickens.

**Results:**

The adjusted prevalence of ESBL-Ec colonization was 20.0% (95% CI 10.8%–29.1%) and 35.2% (95% CI 30.4%–40.1%) in chicken farms and humans in Vietnam, respectively. Colonization with ESBL-Ec in humans was associated with antimicrobial usage (OR = 2.52, 95% CI = 1.08–5.87) but not with involvement in chicken farming. *bla*_CTX-M-55_ was the most common ESBL-encoding gene in strains isolated from chickens (74.4%) compared with *bla*_CTX-M-27_ in human strains (47.0%). In 3 of 204 (1.5%) of the farms, identical ESBL genes were detected in ESBL-Ec isolated from farmers and their chickens. Genomic similarity indicating recent sharing of ESBL-Ec between chickens and farmers was found in only one of these farms.

**Conclusions:**

The integration of epidemiological and genomic data in this study has demonstrated a limited contribution of non-intensive chicken farming to ESBL-Ec colonization in humans in Vietnam and further emphasizes the importance of reducing antimicrobial usage in both human and animal host reservoirs.

## Introduction

The spread of ESBLs in Enterobacteriaceae is a challenge since therapeutic options for infections with these organisms are limited.^1^ ESBL-producing *Escherichia coli* (ESBL-Ec) colonization has been documented in both healthy humans and animals, including chickens,^2^ and the prevalence of ESBL-Ec colonization has increased significantly worldwide.^3^^,^^4^ High and inappropriate antimicrobial drug usage in humans and in animals is an important driving force for this increased prevalence.^5^

It has been suggested that transmission of bacteria and/or mobile genetic elements carrying ESBL-encoding genes from animals to humans may contribute to human infection with ESBL-Ec.^6^ However, recent studies provide partially contradictory conclusions regarding the contribution of poultry to ESBL-Ec colonization and infections in humans. Studies comparing ESBL genes and resistance plasmids in isolates of poultry and human origin suggest that a substantial proportion of human extra-intestinal ESBL-Ec infections may originate from poultry^7–10^ whereas other studies demonstrate that the contribution from poultry is limited.^11^^,^^12^ In addition, these studies mostly compared human *E. coli* isolated from invasive infections with those isolated from purchased chicken meat samples. These isolates were not spatially or temporally associated and the accompanying data on relevant antimicrobial drug usage were lacking. In addition, all of these comparative studies were carried out in high-income countries where industrial well-regulated farming systems are predominant. In contrast, animals in low- and middle-income countries, including Vietnam, are often reared on a small scale with poor biocontainment and unrestricted usage of antimicrobial drugs.^13^^,^^14^ In addition, available data indicate high rates of carriage of antimicrobial-resistant *E. coli* in Vietnam,^15^^,^^16^ a country where antimicrobial drugs for usage in both animals and humans are available over the counter.^17^ However, the risk of human colonization with ESBL-Ec resulting from animal farming has not been addressed. We therefore investigated the contribution of non-intensive chicken farming to ESBL-Ec colonization in humans by determining the prevalence, similarities in resistance encoding gene content, as well as genomic relatedness, of ESBL-Ec isolated from chickens and humans in Vietnam.

## Materials and methods

### Study setting and bacterial isolates

The setting and design of this study have been described previously.^18^ Briefly, a total of 204 chicken farms and 204 chicken farmers were included in our study. In addition, we included two control groups, consisting of 204 individuals from the same districts as the farmers who were not involved in poultry farming, matched by age and gender with the studied farmers (rural controls), and 102 individuals from the provincial city (urban persons).^18^ Data on antimicrobial drug usage in both chickens and humans were collected during medicine cabinet surveys as described previously,^15^ using structured questionnaires (available as Supplementary data at *JAC* Online). Faecal samples including boot swabs from chicken farms and rectal swabs from humans were collected and *E. coli* isolation and identification were performed as described previously.^15^^,^^18^

The presumptive phenotypic production of ESBLs, as indicated by resistance to ceftriaxone and/or ceftazidime was confirmed phenotypically using a double disc diffusion test including ceftriaxone and ceftazidime in the presence and absence of clavulanic acid, in accordance with CLSI guidelines.^19^ A chicken farm or a person was defined as ‘positive’ for ESBL-Ec if at least one ESBL-Ec isolate was detected.

### Data analyses

The adjusted prevalence of faecal colonization with ESBL-Ec in chickens and humans was calculated by assigning a stratum-specific sampling weight to each observation unit (farm or subject) as described previously.^18^

A logistic regression model was built to study risk factors associated with the presence of phenotypically positive ESBL-Ec in humans (Table S1). Variables were included in the multivariate analysis based on a *P* < 0.15 and their biological plausibility of the univariate analyses. ‘Survey’, ‘epicalc’ and ‘adegenet’ packages were used to perform all the statistical analyses using R (https://www.r-project.org/).

**Table 1.** Prevalence of faecal colonization with ESBL-Ec in chickens and humans in southern Vietnam

Subject	No. of ESBL-Ec-positive subjects (prevalence, %)	Adjusted prevalence (95% CI)
Chicken (*N *=* *204)	30 (14.7)	20.0 (10.8–29.1)
small-scale chicken (*N *=* *102)	10 (9.8)	9.4 (2.5–16.3)
household chicken (*N *=* *102)	20 (19.6)	20.0 (10.8–29.3)
Human (*N *=* *510)	205 (40.2)	35.2 (30.4–40.1)
farmer (*N *=* *204)	65 (31.9)	31.1 (24.3–37.8)
rural person (*N *=* *204)	101 (49.5)	47.8 (40.4–55.1)
urban person (*N *=* *102)	39 (38.2)	38.2 (28.7–47.7)

### WGS and phylogenetic analysis

Among 734 ESBL-Ec strains isolated from chickens and humans, to avoid selecting duplicate strains, only one of the identical antimicrobial resistance pattern strains from the same specimen was kept for further analysis. A total of 486 isolates with a unique phenotypic antimicrobial resistance pattern were subjected to WGS. DNA was extracted using the Wizard Genomic DNA purification kit (Promega, Madison, WI, USA) in accordance with the manufacturer’s instructions.

Sequencing was performed at the Wellcome Sanger Institute (UK) using the Illumina Hiseq 2000 (Illumina, Inc., San Diego, CA, USA) with paired-end reads of length 100 bp. An assembly improvement pipeline^20^ using VelvetOptimiser v2.2.5 was conducted to generate *de novo* genome assemblies.^21^ These assemblies were annotated with Prokka^22^ and the output was used for the pan-genome pipeline using Roary^23^ to construct the core gene alignment of 486 ESBL-Ec isolates. Sequence reads were deposited in the European Nucleotide Archive (ENA) and isolate accession numbers are included in Table S6. We identified SNPs in the core gene alignment using an in-house tool (https://github.com/sanger-pathogens/snp-sites). This alignment was used to cluster the isolates into unique subpopulations or sequence clusters using the Bayesian analysis of population structure (hierBAPS).^24^^,^^25^ We also used this alignment to reconstruct the approximately maximum-likelihood tree using FastTree version 2.1.3.^26^ Phylogenetic trees were visualized by using FigTree (http://tree.bio.ed.ac.uk/software/figtree/) and iTOL (v3).^27^

### In silico MLST analysis and identification of antimicrobial resistance determinants

To compare the distribution of ESBL-Ec in our study with other studies, ST was identified for each isolate using the previously developed MLST scheme (http://mlst.warwich.ac.uk/mlst/dbs/Ecoli) with an in-house tool (https://github.com/sanger-pathogens/mlst_check). *In silico* PCR was also used to assign isolates to *E. coli* phylogroups A, B1, B2, C, D, E and F using the Clermont method.^28^

We employed the srst2 package^29^ to identify antimicrobial resistance genes and plasmid incompatibility groups using the ResFinder database^30^ and PlasmidFinder database,^31^ respectively.

We used discriminant analysis of principal components to compare overall acquired antimicrobial resistance gene profiles distribution, not limited to ESBL genes, between the different study groups.^32^

## Results

### Prevalence and risk factors for ESBL-Ec colonization

Among 510 enrolled persons, the median age was 46 (IQR 39–54 years) and 63.9% were male. The prevalence of faecal colonization with ESBL-Ec was significantly higher in humans than chickens (40.2% versus 14.7%, *P *<* *0.001, χ^2^). Among human participants, the prevalence of ESBL-Ec colonization in chicken farmers was significantly lower than in other rural persons (31.9% versus 49.5%, *P *<* *0.001). The adjusted prevalence of ESBL-Ec colonization was 20.0% in chickens, 31.1% in chicken farmers, 47.8% in rural persons and 38.2% in urban persons (Table 1). Rural individuals not involved in poultry farming were at higher risk of colonization with ESBL-Ec than chicken farmers were (OR = 2.04; 95% CI = 1.32–3.15). Usage of antimicrobial drugs in the 4 weeks prior to study participation was associated with human ESBL-Ec colonization (OR = 2.52; 95% CI = 1.08–5.87) (Table 2).

**Table 2.** Multivariate analysis of risk factors associated with faecal colonization with ESBL-Ec in human individuals (*N *=* *510) in southern Vietnam, 2012–13

Variable	No. tested	No. ESBL-Ec positive	OR (95% CI)	*P* value
Participant group				
rural person^a^	204	101	2.04 (1.32–3.15)	0.001
urban person^a^	102	39	1.40 (0.84–2.33)	0.192
farmer	204	65	referent	
Use of any antimicrobial drugs^b^	34	18	2.52 (1.08–5.87)	0.033

Intercept: −0.87 (SEM ± 0.165).

a Not involved in chicken farming.

b During the month prior to the study visit.

### Genetic characterization and phylogeny of ESBL-Ec

The results from MLST indicated that the ESBL-Ec belonged to 85 different STs (Table S2). No ST was assigned to 14 isolates that carried at least one novel allele not included in the database. The most common STs were ST131 (12.8%), ST648 (8.6%), ST38 (7.2%), ST10 (6.4%) and ST69 (5.8%), which together accounted for 40.8% of the total number of isolates. All isolates of the above-mentioned STs, except one, were of human origin. Among 85 STs, 14 (16.5%) were found in both human and chicken host reservoirs (Table S2).

**Figure 1.**
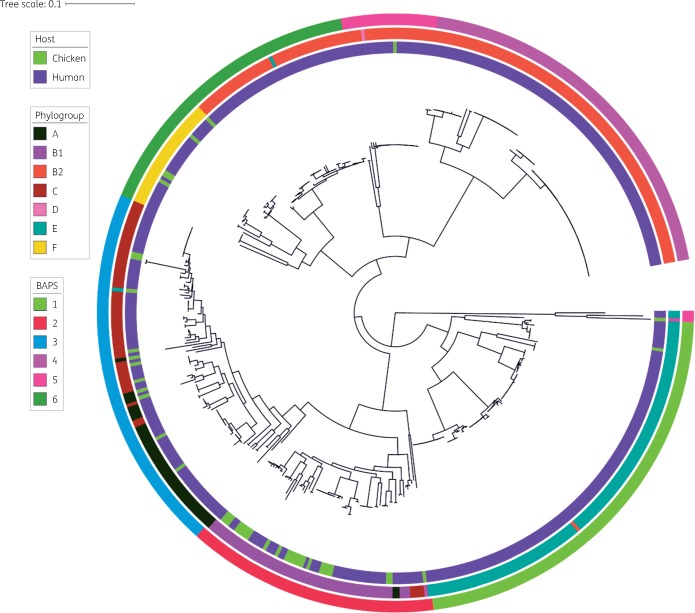
Circular maximum-likelihood core-gene phylogenetic tree of ESBL-Ec isolated from chickens and humans in Vietnam. Phylogenetic tree was reconstructed based on 230 791 SNPs in the core genome of 486 ESBL-Ec isolates. Inner ring designates the host of the isolates. Middle ring designates different phylogroups. Outer ring designates sequence clusters identified by hierBAPS.

A maximum-likelihood phylogenetic tree of the 486 ESBL-Ec was created based on 230 791 SNPs in the core gene alignment, composed of 2232 genes. The core gene phylogeny revealed a diverse population of ESBL-Ec, containing six major lineages, which generally corresponded to the *E. coli* phylogroups except for a few outliers (Figure 1). Chicken and human isolates were mostly intermixed in phylogroups A, B1, C and F. In contrast, the phylogroup B2 was predominantly represented by human isolates.

The population structure of ESBL-Ec isolates was also defined based on BAPS clustering, and the assignment of phylogroups and the output of each method was analysed in the context of the phylogeny (Figure 1). There was a partial concordance between the BAPS clusters and phylogroups, with all isolates from phylogroup B1 assigned to BAPS cluster 2 and the majority of phylogroup E isolates assigned to BAPS cluster 1. Phylogroup A and C were included together in BAPS cluster 3. Similarly, all phylogroup F isolates were included in BAPS cluster 6. In contrast, phylogroup B2 isolates were assigned to three different BAPS clusters (4, 5 and 6). In general, BAPS clustering gave a better definition of ESBL-Ec population structure that was more consistent with the phylogeny, as some phylogroups (e.g. group E) occurred in multiple phylogenetic clusters.

### Distribution of ESBL genes and other resistance genes among ESBL-Ec

**Figure 2.**
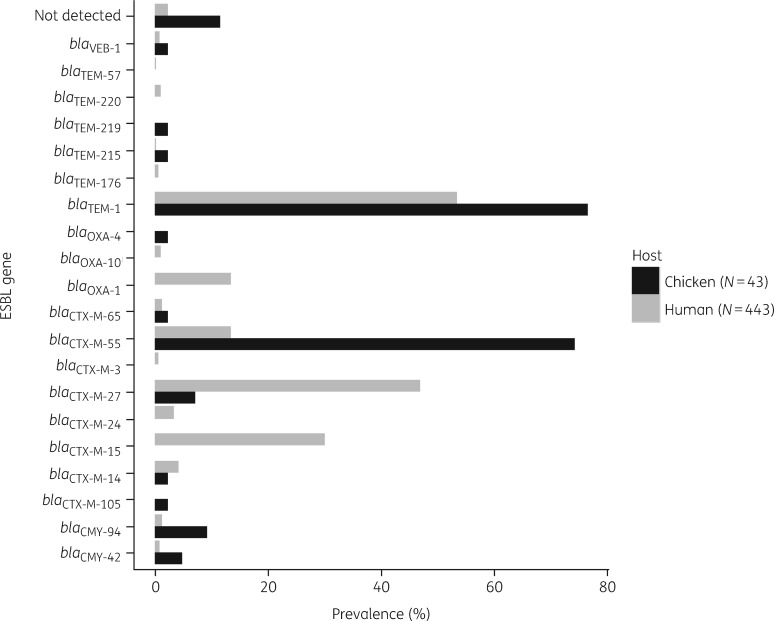
Distribution of β-lactamase genes in 486 ESBL-Ec isolated from chickens and humans in Vietnam.

The distribution of ESBL genes among 486 ESBL-Ec isolated from chickens, farmers and individuals not involved in poultry farming is shown in Figure 2. In general, *bla*_CTX-M_ genes were the predominant ESBL genes, found in 468 of 486 (96.3%) ESBL-Ec isolates. A total of eight subtypes of *bla*_CTX-M_ genes were detected. However, the distribution of *bla*_CTX-M_ gene variants across chicken and human isolates was different. *bla*_CTX-M-55_ was identified as the most common ESBL-encoding gene in chicken isolates (32 of 43, 74.4% versus 60 of 443, 13.5% in human isolates, *P *<* *0.001, χ^2^), whereas *bla*_CTX-M-27_ was the most prevalent in human isolates (208 of 443, 47.0% versus 3 of 43, 7.0% in chicken isolates; *P *<* *0.001, χ^2^). Co-carriage of more than one β-lactamase gene in a single isolate was observed in 312 of 486 (64.2%) isolates.

**Figure 3.**
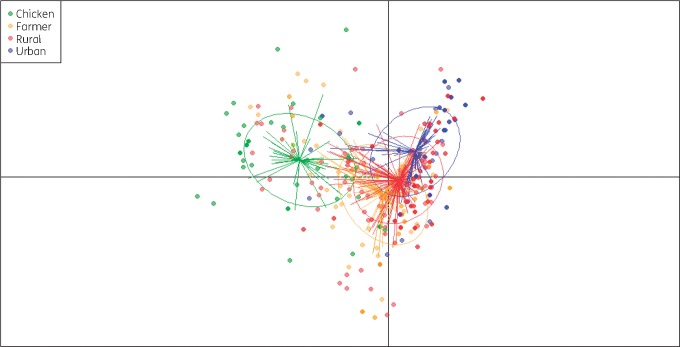
Discriminant analysis of principal components of genotypic antimicrobial resistance profiles of 486 ESBL-Ec isolated from chickens and humans in Vietnam.

The distribution of other antimicrobial resistance genes in ESBL-Ec isolated from farmers, rural individuals and urban individuals is shown in Table S3. The discriminant analysis of principal components of antimicrobial resistance gene profiles, including all detected antimicrobial resistance genes, indicated similarity between isolates from human sources (Figure 3). Isolates from chickens exhibited profiles that were overall distinct from those from the human groups. However, ESBL-Ec in the urban population was most distinct and the antimicrobial resistance gene profiles of the chicken isolates were closer to the rural and farmer isolates than to isolates from the urban population.

**Table 3.** Comparison of ESBL-Ec isolated from farmer and their chickens on the same farm

Farm	Subject	ST	Plasmid replicons	Acquired resistance genes
1	farmer	38	IncFI	*bla*_CTX-M-27_, *aadA5*, *mph*(A), *strA*, *strB*, *sul2*, *tet*(A)
	farmer	38	IncFI	*bla*_CTX-M-27_, *aadA5*, *mph*(A), *strA*, *strB*, *sul2*, *tet*(A)
	chicken	156	IncHI2, IncHI2A, IncN	*bla*_CTX-M-55_, *aac(3)-II*, *aadA2*, *aph(3′)-I*, *bla*_TEM-1_, *dfrA12*, *dfrA14*, *lnu*(F), *mcr-1*, *mph(*A*)*, *strA*, *strB*, *sul2*, *sul3*, *tet*(A)
	chicken	156	ColBS512, IncHI2, IncHI2A, IncN	*bla*_CTX-M-55_, *aac(3)-II*, *aadA2*, *aph(3′)-I*, *bla*_TEM-1_, *dfrA12*, *dfrA14*, *lnu*(F), *mcr-1*, *mph(*A*)*, *strA*, *strB*, *sul2*, *sul3*, *tet*(A)
2	chicken	206	IncFII, IncN	*bla*_CTX-M-55_, *qnrS*, *aadA2*, *bla*_TEM-1_, *cmlA1*, *dfrA12*, *fosA6*, *sul3*, *tet*(A)
	farmer	131	Col156, IncFIA, IncFII, IncI1	*bla*_CTX-M-14_, *aac(3)-II*, *aadA5*, *bla*_TEM-1_, *mph*(A), *strA*, *strB*, *sul2*, *tet*(A)
3	chicken	7160	not detected	*bla*_CTX-M-55_, *qnrS*, *tet*(A)
	farmer	155	IncHI2, IncHI2A	*bla*_CTX-M-55_, *qnrS*, *aac(3)-II*, *aadA5*, *aph(3′)-I*, *bla*_TEM-1_, *dfrA14*, *lnu*(F), *strA*, *strB*, *sul2*, *sul3*, *tet*(A)
	farmer	155	IncFIB, IncHI2, IncHI2A	*bla*_CTX-M-55_, *qnrS*, *aac(3)-II*, *aph(3′)-I*, *bla*_TEM-1_, *dfrA14*, *lnu*(F), *strA*, *strB*, *sul2*, *sul3*, *tet*(A)
4	chicken	617	IncFII, IncQ1, IncR	*bla*_CTX-M-55_, *aac(3)-II*, *aadA2*, *aph(3′)-I*, *aph(3′)-IIa*, *cmlA1*, *dfrA12*, *fosA6*, *sul3*
	chicken	156	not detected	*bla*_CTX-M-55_, *aac(3)-II*, *aac(6′)-Ib-cr*, *bla*_TEM-1_, *catA1*, *catB3*, *dfrA1*, *strA*, *strB*, *sul2*, *tet*(A), *tet*(B)
	chicken	7160	IncY	*bla*_CTX-M-105_, *aac(3)-II*, *aadA2*, *aph(3′)-I*, *bla*_TEM-1_, *strA*, *strB*, *sul2*, *sul3*, *tet*(A)
	farmer	38	IncFIA, IncFII_p	*bla*_CTX-M-27_, *aadA5*, *bla*_TEM-1_, *erm*(B*)*, *mph*(A*)*, *-strA*, *strB*, *sul2*, *tet*(A)
	farmer	38	IncFIA, IncFII_p	*bla*_CTX-M-27_, *aadA5*, *bla*_TEM-1_, *erm*(B*)*, *mph*(A*)*, *-strA*, *strB*, *sul2*, *tet*(A)
	farmer	101	IncFI	*bla*_CTX-M-27_, *aadA2*, *aadA5*, *bla*_TEM-1_, *cmlA1*, *erm*(B), *mcr-1*, *mph*(A)
5	chicken	349	not detected	*bla*_CTX-M-55_, *aac(3)-II*, *dfrA14*, *lnu*(F), *sul3*, *tet*(A)
	farmer	1163	IncFIA	*bla*_CTX-M-27_, *aadA5*, *bla*_TEM-1_, *erm*(B), *mph*(A), *tet*(B)
	farmer	1163	IncFIA	*bla*_CTX-M-27_, *aadA5*, *bla*_TEM-1_, *erm*(B), *mph*(A), *tet*(B)
	farmer	1163	IncFIA	*bla*_CTX-M-27_, *aadA5*, *bla*_TEM-1_, *erm*(B), *mph*(A), *tet*(B)
	farmer	1193	Col156, ColBS512, ColpVC, IncFIA, IncI1	*bla*_CTX-M-15_, *aac(3)-II*, *aadA5*, *bla*_TEM-1_, *mph*(A), *strA*, *strB*, *sul2*, *tet*(A)
6	chicken	448	not detected	*bla*_CTX-M-55_, *aac(3)-II*, *dfrA14*, *sul3*, *tet*(A)
	farmer	131	IncFI	*bla*_CTX-M-24_, *aac(3)-II*, *aadA5*, *bla*_TEM-1_, *mph*(A), *strA*, *strB*, *sul2*, *tet*(A)
7	chicken	457	not detected	*bla*_CTX-M-27_, *aac(3)-II*, *aadA2*, *aph(3′)-I*, *bla*_TEM-1_, *cmlA1*, *dfrA14*, *erm*(B), *strA*, *strB*, *sul2*, *sul3*
	chicken	457	not detected	*bla*_CTX-M-27_, *aac(3)-II*, *aadA2*, *aph(3′)-I*, *bla*_TEM-1_, *cmlA1*, *dfrA14*, *erm*(B), *strA*, *strB*, *sul2*, *sul3*
	farmer	394	IncFI, IncI1	*bla*_CTX-M-15_, *bla*_TEM-1_
	farmer	394	IncFI, IncI1	*bla*_CTX-M-15_, *bla*_TEM-1_
8	chicken	7179	not detected	*bla*_CTX-M-14_, *aac(3)-II*, *aph(3′)-I*, *bla*_TEM-1_, *dfrA14*, *sul2*, *sul3*, *tet*(A)
	farmer	31	IncFIB, IncFII, IncI1	*bla*_CTX-M-27_, *aac(3)-II*, *aadA5*, *bla*_TEM-1_, *catA1*, *erm*(B), *mph*(A), *tet*(A)
	farmer	517	IncFI	*aac(3)-II*, *aadA5*, *bla*_TEM-1_, *mph*(A)
	farmer	31	IncFIB, IncFII, IncI1	*bla*_CTX-M-27_, *aac(3)-II*, *aadA5*, *bla*_TEM-1_, *catA1*, *erm*(B), *mph*(A), *tet*(A)
	farmer	31	IncFIB, IncFII, IncI1	*bla*_CTX-M-27_, *aac(3)-II*, *aadA5*, *bla*_TEM-1_, *catA1*, *erm*(B), *mph*(A), *tet*(A)
9	chicken	101	IncI1, IncR	*bla*_CTX-M-55_, *qnrS*, *aac(3)-II*, *aph(3′)-I*, *bla*_TEM-1_, *dfrA14*, *lnu*(F), *mcr-1*, *sul2*, *sul3*, *tet*(A)
	chicken	101	IncI1, IncR	*bla*_CTX-M-55_, *qnrS*, *aac(3)-II*, *aph(3′)-I*, *bla*_TEM-1_, *dfrA14*, *lnu*(F), *mcr-1*, *sul2*, *sul3*, *tet*(A)
	farmer	7193	IncFI	*bla*_CTX-M-27_, *aadA2*, *dfrA12*, *erm*(B), *mph*(A), *strA*, *strB*, *sul2*, *tet*(A)
10	chicken	6823	IncFI	*aadA5*, *bla*_TEM-1_, *erm*(B), *mph*(A), *strA*, *strB*, *sul2*, *tet*(A)
	chicken	6823	IncFI	*aadA5*, *bla*_TEM-1_, *erm*(B), *mph*(A), *strA*, *strB*, *sul2*, *tet*(A)
	farmer	1193	ColBS512, IncFIA, IncI1	*bla*_CTX-M-15_, *aadA5*, *bla*_TEM-1_, *mph*(A), *strA*, *strB*, *sul2*, *tet*(A)
	farmer	1193	ColBS512, IncFIA, IncI1	*bla*_CTX-M-15_, *aadA5*, *bla*_TEM-1_, *mph*(A), *strA*, *strB*, *sul2*, *tet*(A)
11	chicken	226	IncFIB, IncR	*bla*_CTX-M-65_, *aac(3)-II*, *aadA2*, *cmlA1*, *dfrA12*, *strA*, *strB*, *sul3*, *tet*(A)
	farmer	226	IncFIB, IncR	*bla*_CTX-M-65_, *aac(3)-II*, *aadA2*, *cmlA1*, *dfrA12*, *strA*, *strB*, *sul3*, *tet*(A)
	farmer	unknown	Col8282, ColE10, ColBS512, ColMP18, ColpVC, IncFI, IncFIB, IncQ1, IncR, IncW	*bla*_CTX-M-65_, *qnrS*, *aac(3)-II*, *aadA2*, *aadA5*, *aph(3′)-I*, *bla*_TEM-1_, *cmlA1*, *dfrA12*, *dfrA14*, *strA*, *strB*, *sul3*, *tet*(A)
	farmer	226	IncFIB, IncR	*bla*_CTX-M-65_, *aac(3)-II*, *aadA2*, *cmlA1*, *dfrA12*, *strA*, *strB*, *sul3*, *tet*(A)
12	chicken	746	not detected	*aadA2*, *cmlA1*, *dfrA12*, *sul2*, *sul3*, *tet*(A)
	farmer	69	Col8282	*bla*_CTX-M-27_, *aadA5*, *erm*(B), *mph*(A), *strA*, *strB*, *sul2*, *tet*(A)
	farmer	69	Col8282	*bla*_CTX-M-27_, *aadA5*, *erm*(B), *mph*(A), *strA*, *strB*, *sul2*, *tet*(A)
	farmer	69	Col8282	*bla*_CTX-M-27_, *aadA5*, *erm*(B), *mph*(A), *strA*, *strB*, *sul2*, *tet*(A)
13	farmer	131	not detected	*bla*_CTX-M-15_, *aac(3)-II*, *aac(6′)-Ib-cr*, *aadA5*, *mph*(A), *tet*(A)
	chicken	10	IncA_C	*aadB1*, *bla*_TEM-1_, *catB3*, *dfrA1*, *strA*, *strB*, *sul2*, *tet*(A)
14	chicken	156	not detected	*bla*_CTX-M-55_, *aac(3)-II*, *aph(3′)-I*, *bla*_TEM-1_, *dfrA14*, *lnu*(F), *mcr-1*, *sul3*, *tet*(A)
	farmer	10	ColBS512, IncI1	*bla*_CTX-M-15_, *bla*_TEM-1_, *dfrA1*, *dfrA14*, *mph*(A), *sul2*, *tet*(A)
	farmer	10	ColBS512, IncI1	*bla*_CTX-M-15_, *bla*_TEM-1_, *dfrA1*, *dfrA14*, *mph*(A), *sul2*, *tet*(A)
	farmer	10	ColBS512, IncI1	*bla*_CTX-M-15_, *bla*_TEM-1_, *dfrA1*, *dfrA14*, *mph*(A), *sul2*, *tet*(A)
	farmer	10	ColBS512, IncI1	*bla*_CTX-M-15_, *bla*_TEM-1_, *dfrA1*, *dfrA14*, *mph*(A), *sul2*, *tet*(A)
	farmer	10	ColBS512, IncI1	*bla*_CTX-M-15_, *bla*_TEM-1_, *dfrA1*, *dfrA14*, *mph*(A), *sul2*, *tet*(A)
15	chicken	7200	IncHI2, IncHI2A, IncN	*bla*_CTX-M-55_, *qnrS*, *aac(3)-II*, *aph(3′)-I*, *bla*_TEM-215_, *dfrA14*, *lnu*(F), *mph*(A), *strA*, *strB*, *sul2*, *sul3*
	farmer	226	IncFI, IncFII, IncI1	*bla*_CTX-M-27_, *erm*(B), *mph*(A)
	farmer	226	IncFI, IncFII, IncI1	*bla*_CTX-M-27_, *erm*(B), *mph*(A)
16	chicken	155	IncFIA, IncFIB, IncY	*bla*_CTX-M-55_, *aac(3)-II*, *aadA2*, *aadA5*, *aph(3′)-I*, *aph(4)-Ia*, *bla*_TEM-219_, *cmlA1*, *dfrA12*, *dfrA14*, *lnu*(F), mph(A), *strA*, *strB*, *sul2*, *sul3*, *tet*(A)
	chicken	162	IncFIA, IncY	*bla*_CTX-M-55_, *aac(3)-II*, *aadA2*, *aadA5*, *aph(4)-Ia*, *bla*_TEM-1_, *mph*(A), *strA*, *strB*, *sul2*, *sul3*, *tet*(A)
	farmer	410	not detected	*bla*_CTX-M-55_, *aac(3)-II*, *aph(3′)-I*, *bla*_TEM-1_, *dfrA14*, *strA*, *strB*, *sul2*, *sul3*, *tet*(A)

Underlining indicates that isolates with identical ESBL genes were detected in both the farmer and their chickens on the same farm.

### Plasmid incompatibility groups

**Figure 4.**
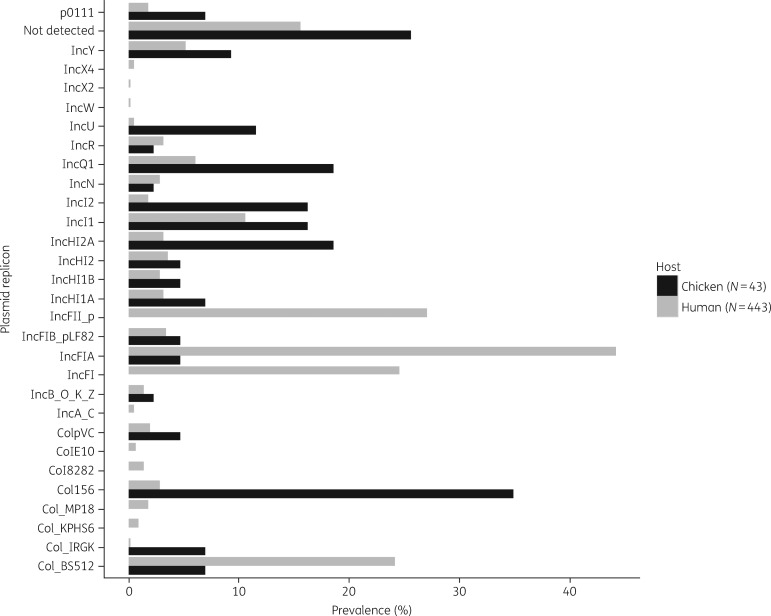
Distribution of plasmid replicons in 486 ESBL-Ec isolated from chickens and humans in Vietnam.

A total of 29 plasmid incompatibility groups were identified by WGS. A total of 19 of 29 (65.5%) plasmid incompatibility groups were presented in ESBL-Ec isolated from both humans and chickens (Figure 4). The most common plasmid incompatibility groups across all 486 ESBL-Ec isolates were IncFIA, IncFII, IncFI, ColBS512, IncI1 and IncHI2A (Figure 4). However, the distribution of plasmid incompatibility groups across chicken and human isolates was different. IncFIA, IncFII, IncFI and ColBS512 were the more common plasmid replicons in human isolates, whereas plasmid replicons Col156, IncHI2A, IncQ1 and IncU were more prevalent in chicken isolates (*P *<* *0.05).

### Characteristics of ESBL-Ec isolated from chickens and farmers on the same farm

On 16 of 204 farms (7.8%; 95% CI = 4.7%–12.7%) ESBL-Ec was detected phenotypically in both the farmers and their chickens on the same farm. WGS revealed that in three farms (1.5%; 95% CI = 0.3%–4.6%), ESBL genes of ESBL-Ec isolated from the farmers and their chickens were identical (farms 3, 11 and 16; Table 3). However, the sequence of single genes provides very limited temporal resolution as even identical single genes can share a long time back to the last common ancestor. We detected identical STs among ESBL-Ec between chicken and farmer isolates in only one farm (farm 11, Table 3). The same plasmid replicon types were observed in this pair of isolates (IncFIB and IncR), although again, temporal resolution is limited with this analysis. The isolates also had similar core gene sequences, with only one pairwise SNP distance based on core gene alignment, indicating potential recent sharing of ESBL-Ec between the chickens and farmer on that farm.

## Discussion

We showed evidence that the contribution of non-intensive chicken farming to ESBL-Ec colonization in occupationally exposed human individuals is very limited in southern Vietnam. Although this study was performed in a non-intensive farming setting, our findings are concordant with recently published studies from Europe.^11^^,^^12^

The prevalence of colonization with ESBL-Ec in chicken farms in Vietnam was 20.0% (95% CI 10.8%–29.1%). This prevalence is relatively low compared with the prevalence of ESBL-Ec colonization in chicken farms in European countries, which can range between 40% and 100%.^33–35^ Although the use of third-generation cephalosporins *in ovo* or in day-old chicks is a probable explanation for the higher prevalence of faecal colonization with ESBL-Ec in industrial chicken farms in these studies in Europe,^36^ such data are not available for Vietnam. Although we found veterinary drugs for chicken usage that contain cephalosporins in a veterinary drug store survey (data not shown), cephalosporin-containing products were not found in the studied chicken farms using the medicine cabinet assessment.^15^ Given the poor biosecurity on the studied chicken farms, ESBL-Ec from other common sources in the farm environment could potentially pass to the chickens as has been demonstrated previously.^37^ In addition, since antimicrobials, such as tetracyclines, streptomycin and sulphonamides were commonly used on these chicken farms,^15^ another plausible explanation for the presence of ESBL-Ec is the co-selection pressure exerted through the use of other classes of antimicrobials.^38^ However, we should interpret these comparisons with caution because the data were obtained in intensive farming settings in Europe as opposed to the household and small-scale farm settings in Vietnam, and used different sampling methods.

In contrast, the prevalence of ESBL-Ec colonization in rural and urban individuals in this study was much higher than reported in Europe^39^ but was similar to the figures reported in the community in other Asian countries.^40^^,^^41^ High ESBL-Ec colonization prevalence is probably the consequence of uncontrolled and high human usage of antimicrobial drugs in the community.^17^ Indeed, although exposure to non-intensive chicken farming did not increase the risk of colonization with ESBL-Ec in humans, we observed an association between human antimicrobial use during the previous month and ESBL-Ec colonization, in line with previous publications in which usage of any class of antimicrobials was identified as one of the risk factors associated with ESBL-Ec colonization.^42^

The diversity of ESBL-Ec in our study was consistent with previous studies.^43^^,^^44^ Although genetically diverse, ST131, ST648, ST38, ST10 and ST69 were the predominant STs. It is interesting to note that these worldwide circulating STs were only detected in human ESBL-Ec isolates, except for one ST10 isolate, which was of chicken origin. In addition, among 204 studied farms, we only detected one pair of chicken–farmer ESBL-Ec isolates on the same farm with identical STs and these had only one pairwise SNP distance based on core gene alignment. These findings suggest a low likelihood that *E. coli* has been recently shared between chickens and humans. The above-mentioned pair of isolates also shared identical plasmid replicons indicating that the dissemination of ESBL genes was most likely due to sharing of the strain together with the plasmid, rather than the plasmid transferring independently of its host strain. Although the frequency of bacterial and/or mobile genetic elements sharing was low (<1.0%), given the high number of household chicken farms in Vietnam (∼7 million), the cumulative burden of exchange of antimicrobial-resistant bacteria and/or their resistance determinants between humans and chickens in the community should not be underestimated.

Although we were not able to identify any carbapenemase-encoding genes in our ESBL-Ec collection, the percentage of isolates that carried the *mcr-1* gene was 4.3% (21 of 486 isolates). Although the co-carriage of ESBL and *mcr-1* in ESBL-Ec was detected in both reservoirs, the proportion was significantly higher in chicken isolates than human isolates (25.6% versus 2.3%, respectively). This adds to the argument that the isolates are not epidemiologically related, as colistin is used only in poultry and not in humans. The data still suggest an alarming trend and given the common presence of ESBL and *mcr-1* genes in *E. coli* isolated from the community in Vietnam and elsewhere,^18^^,^^45^^,^^46^ it is necessary to monitor the acquisition and subsequent transmission of an additional carbapenemase-encoding gene in ESBL-Ec to protect human health.

ESBL-Ec isolates from chickens and humans were not only different in the distribution of ESBL genes but also different in the distribution of plasmid replicons. The findings suggest that different plasmids are potentially circulating in the different host populations, concordant with findings from previous studies.^47^^,^^48^

We are aware of the limitations of a cross-sectional study design, which precludes any inferences on the dynamics of ESBL-Ec sharing between humans and chickens in Vietnam. In addition, the number of isolates from chickens available for WGS was smaller than expected owing to the relatively low prevalence ESBL-Ec colonization in chicken farms. This could have had an important impact on the detection of transmission events where some of them could be missed.^49^ Moreover, we were unable to assemble the plasmid sequences with short read data to infer further the plasmid sequence diversity and compare plasmid distribution between ESBL-Ec from different hosts. In addition, we were unable to identify any known ESBL gene in 15 of 486 ESBL-Ec isolates. As this phenomenon was also described in previous studies,^50^^,^^51^ we have re-checked the phenotype of these isolates and confirmed that 6 of 15 isolates were resistant to third-generation cephalosporins. The remaining nine isolates were susceptible potentially through the loss of ESBL genes/plasmids.^50^^,^^52^ We have removed these nine *E. coli* isolates from the data set and repeated our analyses to ensure that we did not include any ESBL-negative isolates in our prevalence and risk factor analyses. After the analyses, the key messages of the study remained unchanged. These results are presented in Tables S4 and S5. Despite these limitations, this study provides a comprehensive view of the contribution of non-intensive chicken farming to ESBL-Ec colonization in humans in Vietnam where chicken farms and healthy human populations with spatial and temporal association were sampled.

## Supplementary Material

Supplementary Data I

Supplementary Data II
