# Supplementary material for: Limited contribution of non-intensive chicken farming to ESBL-producing Escherichia coli colonization in humans in Vietnam: an epidemiological and genomic analysis
Source: J Antimicrob Chemother. 2019 Jan 9;74(3):561–70. doi: 10.1093/jac/dky506 (PMC6376849; doi:10.1093/jac/dky506)
Supplement: Supplementary Data I [file dky506_supplementary_data_i.doc]

**Supplementary data I**

Text S1: Questionnaire on family medicine use

Name of interviewer: [_________________________________]

Interview date (dd/mm/yy) : [__|__]/[__|__]/[__|__]

We are conducting a study to investigate medicines used by Tien Giang farmers for their chickens and for their own health. You have received information about our study and you have agreed to participate. You gave us information on your medicine use for your chicken. We would now like to ask you some questions about your experience in the use of medicines for your own health and the health of your family members. For example, which medicines do you use, when and why.

Do you agree to do the interview now?

| 1. **GENERAL INFORMATION** | | | | |
| --- | --- | --- | --- | --- |
| 1. Age of respondent: [__|__] | | | | |
| 1. Gender: ⭘ Male ⭘ Female | | | | |
| 1. *This question NOT APPLICABLE for chicken farmers.*   Are there any animal species (not poultry) in your household now? ⭘ Yes ⭘ No  If Yes, tick all that apply  🞏 Pig 🞏 Cattle/buffalo 🞏 Dog 🞏 Cat 🞏 Fish 🞏 Other | | | | |
| 1. Who live in your household? | | | | |
| **No** | **Relationship of family/household member with the respondent**  1. The respondent  2. Respondent’s partner  3. Child (3A for child 1, 3B for child 2 and so on – from oldest to youngest)  4. Mother/Father  5. Grandparents  6. Others (6A. Son/daughter-in-law, 6B. Grandchildren, 6C. other ) | **Age**  (in years, if less than 1 year old, write 01) | **Gender**  1 - Male  2 - Female | **Tick if the person was sampled** |
| 1 | **[_0|1_]** | **[__|__]** | **[__|__]** | [__] |
| 2 | **[__|__]** | **[__|__]** | **[__|__]** | [__] |
| 3 | **[__|__]** | **[__|__]** | **[__|__]** | [__] |
| 4 | **[__|__]** | **[__|__]** | **[__|__]** | [__] |
| 5 | **[__|__]** | **[__|__]** | **[__|__]** | [__] |
| 6 | **[__|__]** | **[__|__]** | **[__|__]** | [__] |
| 7 | **[__|__]** | **[__|__]** | **[__|__]** | [__] |
| 8 | **[__|__]** | **[__|__]** | **[__|__]** | [__] |
| 9 | **[__|__]** | **[__|__]** | **[__|__]** | [__] |
| 10 | **[__|__]** | **[__|__]** | **[__|__]** | [__] |

| 1. MEDICINE REVIEW (ANTIBIOTIC USAGE ONLY) 2. What medicines does your family use to treat illnesses or to stay healthy? Do you keep them in a medicine cabinet? Can we see them?    1. Medicines seen: ⭘ Yes ⭘ No    2. Antibiotics present: ⭘ Yes ⭘ No ⭘ Don’t know    3. Please fill in the following table starting with the antibiotics present in the cabinet, describe them in detail as well as the member in your family treated with those. Then continue listing any other antibiotic not present in the cabinet used by your family member over the last month | | | | | | | | | |
| --- | --- | --- | --- | --- | --- | --- | --- | --- | --- |
| **Antibiotic**  (commercial name) | **Present in the cabinet**  1.Yes  2.No | **Commercial presentation**  (Use code *) | **Active ingredient per unit**  (g or mg) | **Who used the antibiotic**  (Use code**) | **Illness**  (Use code **+**) | **How long ago was the last usage?**  (Use code#) | **How many units per day?** | **Duration**  (How many days) | **Whose advice**  (Use code ++) |
|  | [____] | [__|__] |  | [__|__] | [____] | [____] | [__] | [__|__] | [__] |
|  | [____] | [__|__] |  | [__|__] | [____] | [____] | [__] | [__|__] | [__] |
|  | [____] | [__|__] |  | [__|__] | [____] | [____] | [__] | [__|__] | [__] |
|  | [____] | [__|__] |  | [__|__] | [____] | [____] | [__] | [__|__] | [__] |
|  | [____] | [__|__] |  | [__|__] | [____] | [____] | [__] | [__|__] | [__] |
|  | [____] | [__|__] |  | [__|__] | [____] | [____] | [__] | [__|__] | [__] |
|  | [____] | [__|__] |  | [__|__] | [____] | [____] | [__] | [__|__] | [__] |
|  | [____] | [__|__] |  | [__|__] | [____] | [____] | [__] | [__|__] | [__] |
|  | [____] | [__|__] |  | [__|__] | [____] | [____] | [__] | [__|__] | [__] |
|  |  |  |  |  |  |  |  |  |  |

Codes Lists: *: **T**: Tablet; **C**: capsules; **SY**: syrup; **D**: drops; **I**: injection; **SA**: sachets; **SU**: suppositorium; **P**: pomade (cream)

**: **1**.Respondent, **2**. Respondent’s partner, **3**. Child (3A for child 1, 3B for child 2 and so on – from oldest to youngest), **4**. Parents or Grandparents, **5**. Others

**+**: **R**: Respiratory symptoms/infections; **G**: Gastrointestinal symptoms/infections; **M**: Mouth and teeth symptoms/infections; **S**: wound/skin symptoms/infections; **G**: General malaise symptoms/infections; **O**: other symptoms/infections specify.

#: **1**. still used, **2**. 1-7 days ago, **3**. 1-4 weeks ago, **4**. More than 1 month ago 5. Not use this antibiotic yet

**++**: **1**. Drug sellers; **2**. Doctor/Health professionals; **3**. Friend/neighbor **4**. Others

| 1. **EATING HABITS** |
| --- |
| 1. How often do you eat chicken meat (include any dish with chicken)? (tick 1 only)   ⭘ Everyday or almost every day  ⭘ 3-4 times per week  ⭘ 1-2 times per week  ⭘ 2-3 times per month  ⭘ Less than 2 times per month  ⭘ Never |
| 1. *This question ONLY APPLICABLE for chicken farmers*   Of all chicken meat you eat (include eating out), please choose the statement that better reflects your situation (tick 1 only).  ⭘ All of the chicken meat I eat is not reared in my farm  ⭘ Most of the chicken meat I eat comes from outside and some of it is reared in my farm  ⭘ Some of the chicken meat I eat comes from outside and some of them is reared in my farm  ⭘ Most of the chicken meat I eat comes from my farm and a some of it comes from outside  ⭘ All of the chicken meat I eat comes from my farm |
| 1. How often do you eat chicken eggs (include any dish with eggs)? (tick 1 only)   ⭘ Everyday or almost every day  ⭘ 3-4 times per week  ⭘ 1-2 times per week  ⭘ 2-3 times per month  ⭘ Less than 2 times per month  ⭘ Never |
| 1. *This question ONLY APPLICABLE for chicken farmers*   Of all chicken eggs you eat (include eating out), please choose the statement that better reflects your situation (tick 1 only)  ⭘ All of the chicken eggs I eat come from outside  ⭘ Most of the chicken eggs I eat come from outside and some of them are produced in my farm  ⭘ Some of the chicken eggs I eat come from outside and some of them are produced in my farm  ⭘ Most of the chicken eggs I eat come from my farm and some of them come from outside  ⭘ All of the chicken eggs I eat come from my farm |
| 1. Source of water (tick all that apply)   🞏 Municipal supply 🞏 Borehole/well 🞏 Rain water  🞏 Pond 🞏 River/stream/canal 🞏 other, specify________________   1. Distance to the closest running water sources (in meter) [__|__|__|__|__] |
| 1. **QUESTION** |
| 1. Do you have any questions or comments? ⭘ Yes ⭘ No   [_____________________________________________________________________]  [_____________________________________________________________________] |

Thank you, this is the end of the interview.

**TABLE S1: Univariate analysis of risk factors associated with fecal ESBL-Ec colonization in humans in Vietnam**

| **No** | **Name of variable** | **No. of ESBL (+) subject** | **Total** | **%** | **OR** | **95% CI** | **Pvalue** |
| --- | --- | --- | --- | --- | --- | --- | --- |
| 1 | Participant group |  |  |  |  |  |  |
|  | *Chicken farmers* | 65 | 204 | 31.9% | referent |  |  |
|  | *Rural individual not involved in chicken farming* | 101 | 204 | 49.5% | 2.17 | 1.43 – 3.3 | <0.001 |
|  | *Urban individual not involved in chicken farming* | 39 | 102 | 38.2% | 1.37 | 0.83 – 2.28 | 0.22 |
| 2 | Household location |  |  |  |  |  |  |
|  | *Cho Gao district* | 71 | 170 | 41.8% | 1.4 | 0.89 – 2.22 | 0.147 |
|  | *Chau Thanh district* | 61 | 170 | 35.9% | referent |  |  |
|  | *My Tho city* | 73 | 170 | 42.9% | 1.6 | 1.02 – 2.52 | 0.043 |
| 3 | Age of participant (median age was used as breakpoint) |  |  |  |  |  |  |
|  | *< 46 years old* | 100 | 248 | 40.3% | 0.96 | 1.0 – 1.0 | 0.81 |
|  | *≥ 46 years old* | 105 | 262 | 40.1% | referent |  |  |
| 4 | Male participant | 140 | 326 | 42.9% | 1.48 | 1.0 – 2.2 | 0.048 |
| 5 | Presence of other animals | 122 | 341 | 35.8% | 0.57 | 0.39 – 0.83 | 0.004 |
| 6 | Presence of pig(s) | 32 | 104 | 30.8% | 0.61 | 0.38 – 1.0 | 0.05 |
| 7 | Participants that used cephalosporins in the past month | 11 | 20 | 55.0% | 2.07 | 0.79 – 5.41 | 0.14 |
| 8 | Participants that used antimicrobials in the past month | 18 | 34 | 52.9% | 2.06 | 0.98 – 4.31 | 0.056 |
| 9 | Chicken meat consumption |  |  |  |  |  |  |
|  | *Often (at least twice/week)* | 35 | 91 | 38.5% | 1.1 | 0.44 – 2.75 | 0.84 |
|  | *Sometimes (at least twice/month)* | 158 | 392 | 40.3% | 0.9 | 0.39 – 2.07 | 0.81 |
|  | *Never* | 12 | 27 | 44.4% | referent |  |  |
| 10 | Egg consumption |  |  |  |  |  |  |
|  | *Often (at least twice/week)* | 86 | 222 | 38.7% | 0.9 | 0.44 - 1.82 | 0.76 |
|  | *Sometimes (at least twice/month)* | 101 | 247 | 40.9% | 0.91 | 0.46 – 1.83 | 0.79 |
|  | *Never* | 18 | 41 | 43.9% | referent |  |  |

**TABLE S2** Distribution of STs of ESBL-producing *E. coli* isolated from chickens and humans in Vietnam

| **No** | **ST** | **Chicken (N=43)** | **Human (N=443)** | **Total** |
| --- | --- | --- | --- | --- |
| 1 | 131 |  | 62 | 62 |
| 2 | 648 |  | 42 | 42 |
| 3 | 38 |  | 35 | 35 |
| 4 | 10 | 1 | 30 | 31 |
| 5 | 69 |  | 28 | 28 |
| 6 | 405 |  | 25 | 25 |
| 7 | 1163 | 1 | 19 | 20 |
| 8 | 1193 |  | 19 | 19 |
| 9 | 226 | 1 | 13 | 14 |
| 10 | 354 | 1 | 12 | 13 |
| 11 | 421 |  | 12 | 12 |
| 12 | 156 | 7 | 4 | 11 |
| 13 | 349 | 1 | 10 | 11 |
| 14 | 155 | 5 | 3 | 8 |
| 15 | 457 | 2 | 6 | 8 |
| 16 | Novel ST^a^ | 3 | 4 | 7 |
| 17 | Unknown^b^ | 1 | 6 | 7 |
| 18 | 394 |  | 6 | 6 |
| 19 | 1664 |  | 6 | 6 |
| 20 | 31 |  | 5 | 5 |
| 21 | 62 |  | 5 | 5 |
| 22 | 206 | 1 | 4 | 5 |
| 23 | 448 | 3 | 2 | 5 |
| 24 | 101 | 2 | 2 | 4 |
| 25 | 410 |  | 4 | 4 |
| 26 | 716 |  | 4 | 4 |
| 27 | 3177 |  | 4 | 4 |
| 28 | 48 | 1 | 2 | 3 |
| 29 | 95 |  | 3 | 3 |
| 30 | 196 |  | 3 | 3 |
| 31 | 6756 |  | 3 | 3 |
| 32 | 162 | 1 | 1 | 2 |
| 33 | 165 | 1 | 1 | 2 |
| 34 | 174 |  | 2 | 2 |
| 35 | 205 |  | 2 | 2 |
| 36 | 224 |  | 2 | 2 |
| 37 | 398 |  | 2 | 2 |
| 38 | 453 |  | 2 | 2 |
| 39 | 969 |  | 2 | 2 |
| 40 | 1249 |  | 2 | 2 |
| 41 | 1788 |  | 2 | 2 |
| 42 | 2705 |  | 2 | 2 |
| 43 | 3030 |  | 2 | 2 |
| 44 | 4450 |  | 2 | 2 |
| 45 | 6018 |  | 2 | 2 |
| 46 | 6438 |  | 2 | 2 |
| 47 | 6823 | 2 |  | 2 |
| 48 | 7160 | 2 |  | 2 |
| 49 | 7165 | 2 |  | 2 |
| 50 | 7203 |  | 2 | 2 |
| 51 | 43 |  | 1 | 1 |
| 52 | 59 |  | 1 | 1 |
| 53 | 117 |  | 1 | 1 |
| 54 | 127 |  | 1 | 1 |
| 55 | 175 | 1 |  | 1 |
| 56 | 176 |  | 1 | 1 |
| 57 | 472 |  | 1 | 1 |
| 58 | 484 |  | 1 | 1 |
| 59 | 485 |  | 1 | 1 |
| 60 | 501 |  | 1 | 1 |
| 61 | 517 |  | 1 | 1 |
| 62 | 617 |  | 1 | 1 |
| 63 | 746 | 1 |  | 1 |
| 64 | 773 |  | 1 | 1 |
| 65 | 1295 |  | 1 | 1 |
| 66 | 1485 | 1 |  | 1 |
| 67 | 1638 |  | 1 | 1 |
| 68 | 1722 |  | 1 | 1 |
| 69 | 2064 |  | 1 | 1 |
| 70 | 2220 |  | 1 | 1 |
| 71 | 2914 |  | 1 | 1 |
| 72 | 3014 |  | 1 | 1 |
| 73 | 3489 |  | 1 | 1 |
| 74 | 3580 |  | 1 | 1 |
| 75 | 3910 |  | 1 | 1 |
| 76 | 4553 |  | 1 | 1 |
| 77 | 4985 |  | 1 | 1 |
| 78 | 5073 | 1 |  | 1 |
| 79 | 6395 |  | 1 | 1 |
| 80 | 6616 |  | 1 | 1 |
| 81 | 6734 |  | 1 | 1 |
| 82 | 6761 |  | 1 | 1 |
| 83 | 6769 |  | 1 | 1 |
| 84 | 6780 | 1 |  | 1 |
| 85 | 7193 |  | 1 | 1 |

^a^ the combination of allele numbers has never been seen before

^b^ one of the alleles is not contained in the database

**TABLE S3** Distribution of acquired antimicrobial resistance genes, not limited to ESBL genes, of ESBL –producing *E. coli* isolated from chickens and humans in Vietnam

| **AMR genes** | **Chicken (N=43)** | **Human (N=443)** | **Test stat.** | **P value** |
| --- | --- | --- | --- | --- |
| *qnrB* | 0/43 (0%) | 3/443 (0.7%) | Fisher's exact test | 1 |
| *qnrS* | 15/43 (34.9%) | 59/443 (13.3%) | Chisq. (1 df) = 12.5 | < 0.001 |
| *qnrVC* | 1/43 (2.3%) | 2/443 (0.5%) | Fisher's exact test | 0.243 |
| *aac(3)-II* | 26/43 (60.5%) | 217/443 (49%) | Chisq. (1 df) = 1.63 | 0.201 |
| *aac(3)-VIa* | 0/43 (0%) | 5/443 (1.1%) | Fisher's exact test | 1 |
| *aac(6’)-Ib-cr* | 2/43 (4.7%) | 61/443 (13.8%) | Chisq. (1 df) = 2.14 | 0.144 |
| *aadA1* | 25/43 (58.1%) | 80/443 (18.1%) | Chisq. (1 df) = 34.85 | < 0.001 |
| *aadA2* | 16/43 (37.2%) | 59/443 (13.3%) | Chisq. (1 df) = 15.36 | < 0.001 |
| *aadA5* | 10/43 (23.3%) | 232/443 (52.4%) | Chisq. (1 df) = 12.15 | < 0.001 |
| *aadB1* | 2/43 (4.7%) | 11/443 (2.5%) | Fisher's exact test | 0.322 |
| *aph(3’)-I* | 21/43 (48.8%) | 50/443 (11.3%) | Chisq. (1 df) = 41.34 | < 0.001 |
| *aph(3’)-IIa* | 1/43 (2.3%) | 2/443 (0.5%) | Fisher's exact test | 0.243 |
| *aph(4)-Ia* | 5/43 (11.6%) | 3/443 (0.7%) | Fisher's exact test | < 0.001 |
| *catA1* | 2/43 (4.7%) | 52/443 (11.7%) | Fisher's exact test | 0.207 |
| *catB3* | 2/43 (4.7%) | 1/443 (0.2%) | Fisher's exact test | 0.022 |
| *cmlA1* | 13/43 (30.2%) | 49/443 (11.1%) | Chisq. (1 df) = 11.28 | < 0.001 |
| *dfrA17* | 10/43 (23.3%) | 253/443 (57.1%) | Chisq. (1 df) = 16.75 | < 0.001 |
| *dfrA5* | 1/43 (2.3%) | 12/443 (2.7%) | Fisher's exact test | 1 |
| *dfrA1* | 2/43 (4.7%) | 22/443 (5.0%) | Fisher's exact test | 1 |
| *dfrA12* | 8/43 (18.6%) | 53/443 (12.0%) | Chisq. (1 df) = 1.03 | 0.311 |
| *dfrA14* | 28/43 (65.1%) | 70/443 (15.8%) | Chisq. (1 df) = 56.19 | < 0.001 |
| *erm*(B) | 6/43 (14.0%) | 182/443 (41.1%) | Chisq. (1 df) = 11.05 | < 0.001 |
| *fosA6* | 3/43 (7.0%) | 7/443 (1.6%) | Fisher's exact test | 0.05 |
| *lnu*(F) | 21/43 (48.8%) | 21/443 (4.7%) | Fisher's exact test | < 0.001 |
| *mcr-1* | 11/43 (25.6%) | 10/443 (2.3%) | Fisher's exact test | < 0.001 |
| *mph*(A) | 12/43 (27.9%) | 312/443 (70.4%) | Chisq. (1 df) = 30.01 | < 0.001 |
| *strA* | 24/43 (55.8%) | 241/443 (54.4%) | Chisq. (1 df) = 0 | 0.986 |
| *strB* | 25/43 (58.1%) | 230/443 (51.9%) | Chisq. (1 df) = 0.38 | 0.535 |
| *sul1* | 10/43 (23.3%) | 271/443 (61.2%) | Chisq. (1 df) = 21.58 | < 0.001 |
| *sul2* | 28/43 (65.1%) | 255/443 (57.6%) | Chisq. (1 df) = 0.64 | 0.425 |
| *sul3* | 30/43 (69.8%) | 72/443 (16.3%) | Chisq. (1 df) = 64.5 | < 0.001 |
| *tet*(A) | 36/43 (83.7%) | 263/443 (59.4%) | Chisq. (1 df) = 8.82 | 0.003 |
| *tet*(B) | 3/43 (7.0%) | 122/443 (27.5%) | Chisq. (1 df) = 7.63 | 0.006 |
| *bla*_CTX-M-27_ | 3/43 (7.0%) | 208/443 (47.0%) | Chisq. (1 df) = 23.9 | < 0.001 |
| *bla*_CTX-M-15_ | 0/43 (0%) | 133/443 (30%) | Chisq. (1 df) = 16.3 | < 0.001 |
| *bla*_CTX-M-55_ | 32/43 (74.4%) | 60/443 (13.5%) | Chisq. (1 df) = 90.72 | < 0.001 |
| *bla*_CTX-M-14_ | 1/43 (2.3%) | 18/443 (4.1%) | Fisher's exact test | 1 |
| *bla*_CTX-M-24_ | 0/43 (0%) | 15/443 (3.4%) | Fisher's exact test | 0.383 |
| *bla*_CTX-M-65_ | 1/43 (2.3%) | 5/443 (1.1%) | Fisher's exact test | 0.428 |
| *bla*_CTX-M-3_ | 0/43 (0%) | 2/443 (0.5%) | Fisher's exact test | 1 |
| *bla*_CTX-M-105_ | 1/43 (2.3%) | 0/443 (0%) | Fisher's exact test | 0.088 |
| *bla*_VEB-1_ | 1/43 (2.3%) | 3/443 (0.7%) | Fisher's exact test | 0.31 |
| *bla*_OXA-10_ | 0/43 (0%) | 4/443 (0.9%) | Fisher's exact test | 1 |
| *bla*_OXA-1_ | 0/43 (0%) | 60/443 (13.5) | Chisq. (1 df) = 5.45 | 0.02 |
| *bla*_OXA-4_ | 1/43 (2.3%) | 0/443 (0%) | Fisher's exact test | 0.088 |
| *bla*_TEM-1_ | 33/43 (76.7%) | 237/443 (53.5%) | Chisq. (1 df) = 7.66 | 0.006 |
| *bla*_TEM-176_ | 0/43 (0%) | 2/443 (0.5%) | Fisher's exact test | 1 |
| *bla*_TEM-215_ | 1/43 (2.3%) | 1/443 (0.2%) | Fisher's exact test | 0.169 |
| *bla*_TEM-219_ | 1/43 (2.3%) | 0/443 (0%) | Fisher's exact test | 0.088 |
| *bla*_TEM-220_ | 0/43 (0%) | 4/443 (0.9%) | Fisher's exact test | 1 |
| *bla*_TEM-57_ | 0/43 (0%) | 1/443 (0.2%) | Fisher's exact test | 1 |
| *bla*_CMY-42_ | 2/43 (4.7%) | 3/443 (0.7%) | Fisher's exact test | 0.064 |
| *bla*_CMY-94_ | 4/43 (9.3%) | 5/443 (1.1%) | Fisher's exact test | 0.005 |

**TABLE S4** Prevalence of ESBL-producing *Escherichia coli* colonization in humans in Vietnam after removing 9 ESBL negative *E. coli* isolates.

| **Subject** | **No. of ESBL-Ec positive subjects (Prevalence; %)** | **Adjusted Prevalence (95% CI)** |
| --- | --- | --- |
| Chicken (N=204) | 27 (13.2) | 19.5 (10.4 – 28.7) |
| *Small-scale chicken (N=102)* | 9 (8.8) | 8.9 (2.0 – 15.7) |
| *Household chicken (N=102)* | 18 (17.6) | 19.6 (10.4 – 28.9) |
| Human (N=510) | 203 (39.8) | 34.2 (29.3 – 39.0) |
| *Farmer (N=204)* | 64 (31.4) | 30.4 (23.7 – 37.1) |
| *Rural individual (N=204)* | 100 (49.0) | 47.2 (39.9 – 54.6) |
| *Urban individual (N=102)* | 39 (38.2) | 38.2 (28.8 – 47.7) |

**TABLE S5** Risk factors associated with ESBL-producing *Escherichia coli* colonization in humans in Vietnam after removing 9 ESBL negative *E. coli* isolates.

| **Variables** | **No. tested** | **No. ESBL-Ec positive** | **OR (95% CI)** | **p-value** |
| --- | --- | --- | --- | --- |
| Participant group |  |  |  |  |
| Rural person^a^ | 204 | 100 | 2.07 (1.33 – 3.20) | 0.001 |
| Urban person^a^ | 102 | 39 | 1.45 (0.87 – 2.42) | 0.151 |
| Farmer | 204 | 64 | Referent |  |
| Use of any antimicrobial drugs^b^ | 34 | 18 | 2.59 (1.11 – 6.04) | 0.028 |

Intercept: - 0.90 (SEM±0.166)

^a^not involved in chicken farming

^b^during the month prior to the study visit
