# Supplementary material for: Limited contribution of non-intensive chicken farming to ESBL-producing Escherichia coli colonization in humans in Vietnam: an epidemiological and genomic analysis
Source: J Antimicrob Chemother. 2019 Jan 9;74(3):561–70. doi: 10.1093/jac/dky506 (PMC6376849; doi:10.1093/jac/dky506)
Supplement: Supplementary Data II [file dky506_supplementary_data_ii.doc]

Supplementary data II

**Table S6.** European Nucleotide Accession Numbers of 486 sequenced isolates

No ENA accession ID

1 ERS1152573

2 ERS1152691

3 ERS1151906

4 ERS1152018

5 ERS1152129

6 ERS1152673

7 ERS1152825

8 ERS1152833

9 ERS1152835

10 ERS1152837

11 ERS1152851

12 ERS1152853

13 ERS1152859

14 ERS1151848

15 ERS1151881

16 ERS1152000

17 ERS1152060

18 ERS1152125

19 ERS1152140

20 ERS1152166

21 ERS1152176

22 ERS1152189

23 ERS1152326

24 ERS1152339

25 ERS1152373

26 ERS1152380

27 ERS1152395

28 ERS1152701

No ENA accession ID

29 ERS1152727

30 ERS1152733

31 ERS1151966

32 ERS1152186

33 ERS1152198

34 ERS1152422

35 ERS1152469

36 ERS1152481

37 ERS1152502

38 ERS1152647

39 ERS1151843

40 ERS1151854

41 ERS1152109

42 ERS1152149

43 ERS1152172

44 ERS1152665

45 ERS1152698

46 ERS1151699

47 ERS1151736

48 ERS1151836

49 ERS1151847

50 ERS1151925

51 ERS1151935

52 ERS1151984

53 ERS1151992

54 ERS1152146

55 ERS1152195

56 ERS1152232

No ENA accession ID

57 ERS1152272

58 ERS1152295

59 ERS1152328

60 ERS1152464

61 ERS1152537

62 ERS1151983

63 ERS1152010

64 ERS1152053

65 ERS1152097

66 ERS1152544

67 ERS1152595

68 ERS1152792

69 ERS1152822

70 ERS1152824

71 ERS1152495

72 ERS1152503

73 ERS1152513

74 ERS1152522

75 ERS1152535

76 ERS1152549

77 ERS1152583

78 ERS1152618

79 ERS1152628

80 ERS1152685

81 ERS1151667

82 ERS1151677

83 ERS1151692

84 ERS1151876

No ENA accession ID

85 ERS1152007

86 ERS1152161

87 ERS1152588

88 ERS1152603

89 ERS1152613

90 ERS1152671

91 ERS1151862

92 ERS1152025

93 ERS1152860

94 ERS1152862

95 ERS1152882

96 ERS1152885

97 ERS1152887

98 ERS1152889

99 ERS1152890

100 ERS1152892

101 ERS1152894

102 ERS1152896

103 ERS1152898

104 ERS1152900

105 ERS1152904

106 ERS1151672

107 ERS1151681

108 ERS1151690

109 ERS1151702

110 ERS1151821

111 ERS1152212

112 ERS1152223

113 ERS1152356

114 ERS1152425

115 ERS1152467

116 ERS1152497

117 ERS1151704

118 ERS1151719

119 ERS1151748

120 ERS1151861

121 ERS1152102

122 ERS1152388

123 ERS1152531

124 ERS1152542

125 ERS1152558

126 ERS1152572

127 ERS1152615

128 ERS1152629

129 ERS1152643

130 ERS1152661

131 ERS1152721

132 ERS1152758

133 ERS1151747

134 ERS1151755

135 ERS1151766

136 ERS1151894

137 ERS1151905

138 ERS1151917

139 ERS1151939

140 ERS1151952

141 ERS1151979

142 ERS1152099

143 ERS1152116

144 ERS1152127

145 ERS1152134

146 ERS1152142

147 ERS1152151

148 ERS1152239

No ENA accession ID

149 ERS1152290

150 ERS1152317

151 ERS1152385

152 ERS1152440

153 ERS1152518

154 ERS1152529

155 ERS1152679

156 ERS1151717

157 ERS1151785

158 ERS1151795

159 ERS1151807

160 ERS1152219

161 ERS1152258

162 ERS1152354

163 ERS1152367

164 ERS1152384

165 ERS1152398

166 ERS1152438

167 ERS1152591

168 ERS1152927

169 ERS1152941

170 ERS1152946

171 ERS1152948

172 ERS1152949

173 ERS1152951

174 ERS1153075

175 ERS1153081

176 ERS1153220

177 ERS1153225

178 ERS1153321

179 ERS1153327

180 ERS1152964

181 ERS1153011

182 ERS1153047

183 ERS1153053

184 ERS1153086

185 ERS1153149

186 ERS1153266

187 ERS1153277

188 ERS1153282

189 ERS1152971

190 ERS1153135

191 ERS1153146

192 ERS1153151

193 ERS1153261

194 ERS1153268

195 ERS1153296

196 ERS1153304

197 ERS1153409

198 ERS1152978

199 ERS1153074

200 ERS1153112

201 ERS1153234

202 ERS1153242

203 ERS1153259

204 ERS1153332

205 ERS1153339

206 ERS1153384

207 ERS1153393

208 ERS1153003

209 ERS1153008

210 ERS1153025

211 ERS1153093

212 ERS1153105

No ENA accession ID

213 ERS1153127

214 ERS1153142

215 ERS1153150

216 ERS1153221

217 ERS1153226

218 ERS1153258

219 ERS1153264

220 ERS1153400

221 ERS1153036

222 ERS1153043

223 ERS1153097

224 ERS1153102

225 ERS1153108

226 ERS1153113

227 ERS1153163

228 ERS1153170

229 ERS1153175

230 ERS1153252

231 ERS1153256

232 ERS1153290

233 ERS1153308

234 ERS1153362

235 ERS1153368

236 ERS1153374

237 ERS1153381

238 ERS1153392

239 ERS1153396

240 ERS1153402

241 ERS1153439

242 ERS1232378

243 ERS1232385

244 ERS1232399

245 ERS1232402

246 ERS1232405

247 ERS1232421

248 ERS1232432

249 ERS1232445

250 ERS1232448

251 ERS1232458

252 ERS1232537

253 ERS1232350

254 ERS1232352

255 ERS1232381

256 ERS1232437

257 ERS1232465

258 ERS1232489

259 ERS1232428

260 ERS1232436

261 ERS1232455

262 ERS1232479

263 ERS1232485

264 ERS1232500

265 ERS1232567

266 ERS1232620

267 ERS1232639

268 ERS1232653

269 ERS1232706

270 ERS1232708

271 ERS1232486

272 ERS1232497

273 ERS1232538

274 ERS1232561

275 ERS1232614

No ENA accession ID

276 ERS1232637

277 ERS1232646

278 ERS1232685

279 ERS1232691

280 ERS1232694

281 ERS1232698

282 ERS1232701

283 ERS1232704

284 ERS1232736

285 ERS1232752

286 ERS1232762

287 ERS1232763

288 ERS1233237

289 ERS1233241

290 ERS1233247

291 ERS1233252

292 ERS1233257

293 ERS1233262

294 ERS1233266

295 ERS1233282

296 ERS1233292

297 ERS1233300

298 ERS1233306

299 ERS1232894

300 ERS1232900

301 ERS1233045

302 ERS1233094

303 ERS1233101

304 ERS1233110

305 ERS1233120

306 ERS1233143

307 ERS1233172

308 ERS1233180

309 ERS1233193

310 ERS1233198

311 ERS1232921

312 ERS1232925

313 ERS1232930

314 ERS1232988

315 ERS1232992

316 ERS1232997

317 ERS1233001

318 ERS1233006

319 ERS1233096

320 ERS1233145

321 ERS1233208

322 ERS1233212

323 ERS1233216

324 ERS1233227

325 ERS1233442

326 ERS1233447

327 ERS1233048

328 ERS1233214

329 ERS1233303

330 ERS1233311

331 ERS1233341

332 ERS1233347

333 ERS1233353

334 ERS1233362

335 ERS1233419

336 ERS1233435

337 ERS1233455

338 ERS1233459

339 ERS1233461

No ENA accession ID

340 ERS1233520

341 ERS1233524

342 ERS1233551

343 ERS1233555

344 ERS1233060

345 ERS1233148

346 ERS1233155

347 ERS1233162

348 ERS1233170

349 ERS1233177

350 ERS1233206

351 ERS1233213

352 ERS1233312

353 ERS1233318

354 ERS1233323

355 ERS1233328

356 ERS1233339

357 ERS1233345

358 ERS1233349

359 ERS1233432

360 ERS1233436

361 ERS1233441

362 ERS1233445

363 ERS1233450

364 ERS1232487

365 ERS1232514

366 ERS1232535

367 ERS1232539

368 ERS1232548

369 ERS1232551

370 ERS1232555

371 ERS1232559

372 ERS1232613

373 ERS1232617

374 ERS1232621

375 ERS1232626

376 ERS1232699

377 ERS1232702

378 ERS1232709

379 ERS1232782

380 ERS1232806

381 ERS1232826

382 ERS1232833

383 ERS1232835

384 ERS1232901

385 ERS1232905

386 ERS1232920

387 ERS1232924

388 ERS1232936

389 ERS1233095

390 ERS1233174

391 ERS1233183

392 ERS1233190

393 ERS1233196

394 ERS1233202

395 ERS1233239

396 ERS1232803

397 ERS1232805

398 ERS1232825

399 ERS1232848

400 ERS1232876

401 ERS1232891

402 ERS1232895

No ENA accession ID

403 ERS1232899

404 ERS1232912

405 ERS1232916

406 ERS1232950

407 ERS1232970

408 ERS1232999

409 ERS1233004

410 ERS1233028

411 ERS1233038

412 ERS1233044

413 ERS1233054

414 ERS1233062

415 ERS1233160

416 ERS1232845

417 ERS1232875

418 ERS1232878

419 ERS1232882

420 ERS1232885

421 ERS1232914

422 ERS1232917

423 ERS1232928

424 ERS1232933

425 ERS1232938

426 ERS1232944

427 ERS1232953

428 ERS1232959

429 ERS1233003

430 ERS1233017

431 ERS1233036

432 ERS1233040

433 ERS1233159

434 ERS1233200

435 ERS1233204

436 ERS1233512

437 ERS1233517

438 ERS1233521

439 ERS1233523

440 ERS1233529

441 ERS1233532

442 ERS1233540

443 ERS1233545

444 ERS1233560

445 ERS1233562

446 ERS1233223

447 ERS1233259

448 ERS1233272

449 ERS1233284

450 ERS1233305

451 ERS1233424

452 ERS1233434

453 ERS1233541

454 ERS1233661

455 ERS1233663

456 ERS1233679

457 ERS1233703

458 ERS1233298

459 ERS1233307

460 ERS1233343

461 ERS1233496

462 ERS1233503

463 ERS1233508

464 ERS1233514

465 ERS1233525

No ENA accession ID

466 ERS1233531

467 ERS1233586

468 ERS1233598

469 ERS1233608

470 ERS1233638

471 ERS1233642

472 ERS1233667

473 ERS1233706

474 ERS1233708

475 ERS1233709

476 ERS1233710

477 ERS1233579

478 ERS1233582

479 ERS1233605

480 ERS1233606

481 ERS1233610

482 ERS1233613

483 ERS1233618

484 ERS1233623

485 ERS1233632

486 ERS1233648
